# The PROB-PD trial: a pilot, randomised, placebo-controlled study protocol to evaluate the feasibility and potential efficacy of probiotics in modulating peripheral immunity in subjects with Parkinson’s disease

**DOI:** 10.1186/s40814-023-01306-1

**Published:** 2023-05-08

**Authors:** Stefano Martini, Franca Marino, Luca Magistrelli, Elena Contaldi, Marco Cosentino, Cristoforo Comi

**Affiliations:** 1 Center for Research in Medical Pharmacology, University of Insubria, Via Monte Generoso n. 71, 21100 Varese, VA Italy; 2 Center for Research in Neuroscience, University of Insubria, Via Manara n. 7, Busto Arsizio, VA 21052 Italy; 3 PhD Program in Clinical and Experimental Medicine and Medical Humanities, University of Insubria, Varese, Italy; 4 Movement Disorders Centre, Neurology Unit, Department of Translational Medicine, University of Piemonte Orientale, Novara, Italy; 5 PhD Program in Medical Sciences and Biotechnology, University of Piemonte Orientale, Novara, Italy

**Keywords:** Parkinson’s disease, Probiotics, Inflammation, Peripheral immunity, Innate immunity

## Abstract

**Background:**

Parkinson’s disease (PD) is a common neurodegenerative disease. No disease-modifying treatment is available, and therapy is symptomatic. The histopathologic hallmark is the loss of dopaminergic neurons and accumulation of α-synuclein (α-syn) in surviving neurons, but the underlying pathophysiology is unclear. Inflammatory mechanisms seem to play a prominent role, with an imbalance of immune functions and neurotoxicity caused by reactive oxygen species (ROS). Involvement of peripheral adaptive immunity, with an imbalance in T cell subpopulations and in the expression of transcriptional factors in CD4+ T cells, has also been reported. Although clinical presentation is defined by motor symptoms, patients also report non-motor symptoms, often before the onset of a clinically established disease. Etiopathogenesis of PD is unknown, but an initial aggregation of α-syn in the gut, with subsequent propagation along the vagus nerve to the brain has been hypothesised. Interestingly, in an α-syn overexpressing murine model, the absence of gut microbiota prevented both microglia activation and motor impairment, thus pointing to a fundamental role of microbiota in the development of PD. Magistrelli et al. showed that in peripheral blood mononuclear cells of PD patients, probiotics modulate the in vitro production of cytokines toward an anti-inflammatory profile and reduce the production of ROS.

**Methods:**

This is a pilot randomised placebo-controlled clinical trial protocol for a 12-week treatment with probiotics. At least 80 patients affected by PD will be recruited and randomly allocated to either the treatment or placebo group in a 1:1 ratio. General inclusion criteria will be the onset of PD 2 to 5 years before the trial and absence of autoimmune comorbidities or immunomodulating therapy. Our primary endpoint is the assessment of changes in extracellular cytokine levels (Interferon (IFN)-γ, tumour necrosis factor (TNF)-α, interleukin (IL)-4, and IL-10) and ROS production. Secondary outcomes include changes in lymphocyte subpopulations and transcriptional factors mRNA levels.

**Discussion:**

This study is designed to highlight the potential beneficial role of probiotics administration on peripheral immunity through the modulation of gut microbiota. Explorative outcomes will be evaluated to assess variations in motor and non-motor symptoms and the possible correlation with probiotics administration.

**Trial registration:**

ClinicalTrials.gov ID NCT05173701. Registered 08 November 2021

## Introduction

### Background and rationale

Parkinson’s disease (PD) is a common neurodegenerative disease, affecting up to 1–2 people in 1000. Prevalence increases with age and is estimated at 1% in people over 65 [1]. No treatment which prevents PD onset or delays its progression is yet available, and therapy is focused on the symptom management through dopamine supplementation and the use of direct and indirect dopaminergic agents [2]. The histopathologic hallmark of PD is the loss of dopaminergic neurons and accumulation of α-synuclein (α-syn) in surviving neurons, but the underlying pathophysiology is still unclear [3]. Inflammatory mechanisms have been suggested to play a prominent role in the development of the disease [4], with an imbalance of immune functions, as well as neurotoxicity caused by reactive oxygen species (ROS) [5, 6].

Further evidence highlights the involvement of peripheral adaptive immunity in PD [7, 8], reporting an imbalance in T cell subpopulations and in the expression of transcriptional factors in CD4+ T cells [9] in PD patients and in subjects with REM sleep behaviour disorder, which is considered a potential prodromal state in the development of PD [10]. Although clinical presentation is defined by the presence of motor symptoms such as bradykinesia, rest tremor, and rigidity, patients often also report non-motor symptoms including hyposmia, constipation, pain, anxiety, and depression that may precede the onset of a clinically established disease [11, 12].

Etiopathogenesis of PD is unknown, but work by Braak et al. hypothesised an initial aggregation of α-syn in the gut with subsequent propagation along the vagus nerve to the brain, finally reaching the substantia nigra in the mesencephalon [13].

Interestingly, in an α-syn overexpressing murine model of PD, the absence of gut microbiota prevented both microglia activation and motor impairment [14], thus pointing to a fundamental role of the gut and microbiota in the pathogenesis and development of PD.

In a recent paper, Magistrelli et al. showed that in peripheral blood mononuclear cells (PMBCs) of a cohort of PD patients, probiotics were able to modulate the production of cytokines toward an anti-inflammatory profile and to reduce the production of reactive oxygen species (ROS) [15]. The clinical effects of probiotics have also been explored in other pathological conditions. Tankou et al. administered a probiotic mixture containing viable lyophilized bacteria including four strains of Lactobacillus and three strains of Bifidobacterium in nine multiple sclerosis patients, whose peripheral immune system shifted towards an anti-inflammatory profile, with an inverse tendency after administration discontinuation [16]. These results were confirmed by the same group in a different trial [17].

In the light of this evidence, we designed a pilot randomised placebo-controlled clinical trial to test the potential of probiotics therapy in modulating the inflammatory phenotype, to possibly normalise the gut-brain-microbiota axis, decreasing peripheral inflammation to finally affect disease progression in a cohort of PD patients. Feasibility objectives will be evaluated in the pilot clinical trial, including recruitment, acceptance, and dropout rates. The measurement of possible changes in intracellular and extracellular cytokine levels (IFN-γ, TNF-α, IL-4, and IL-10), ROS production, transcriptional factors mRNA levels, and lymphocyte subpopulations will be used for the selection of appropriate primary outcomes for the following main trial.

#### Objectives

The aim of this pilot study protocol is to assess the feasibility of a randomised controlled trial to assess the effects of a 3-month probiotics therapy in a cohort of Parkinson’s disease patients. Specific feasibility objectives will be assessed to define the feasibility of the proposed clinical trial, including recruitment, acceptance and dropout rate, and outcome identification, as listed in the following Table 1.

**Table 1:** Feasibility objectives

**Objective**	**Description**
Recruitment rate	The number of identified eligible subjects in a given amount of time, at the proposed clinical setting.
Acceptance rate	The fraction of identified eligible subjects who will be willing to participate in the proposed clinical trial.
Dropout rate and safety	Dropout rate will be reported as the fraction of enrolled subjects who will be dropped from the study for any reason. Reported reasons for subject drop-out will be recorded and analysed to confirm safety for both the treatment and placebo administration to enrolled subjects.
Primary outcome identification and sample size estimate	The primary outcome for the future main clinical trial will be identified among significant immune profile changes detected in the pilot trial. Data from the pilot trial will also be used to estimate an appropriate sample size for the main trial.
Secondary outcomes identification	Collected data in the pilot trial will be used to identify promising secondary outcome measures.

Data collected during this pilot study will include the following assessments to evaluate possible outcome measures for the definitive randomised controlled trial:

- 1) Cytokines production (IFN-γ, TNF-α, IL-4, IL-10), as already tested in vitro, to confirm the immune-modulating potential of probiotics.
- 2) Assessment of CD4+ and CD8+ T naïve/memory cells, CD4+ T helper subsets, CD4+ regulatory T cells, monocytes, and NK cells to test the immunomodulating effects of probiotics administration on the immune profile.
- 3) Monitoring of motor and non-motor symptoms of PD, including gut function, and subjective quality of life during the trial period.

#### Trial design

This pilot randomised, double-blind, placebo-controlled trial will recruit Parkinson’s disease patients. This is a prospective, exploratory, interventional study. Enrolled subjects will be equally randomised (1:1) in the placebo or intervention group.

## Methods: Participants, interventions, and outcomes

### Study setting

Patients will be enrolled at Azienda Ospedaliero-Universitaria Maggiore della Carità di Novara, where all visits will be scheduled. The setting of the study is a centre of care and research and an academic hospital.

### Eligibility criteria

All included patients will have a diagnosis of Parkinson’s disease with a disease duration between 2 and 5 years at baseline. Exclusion criteria for this study are listed in the following Table 2:

**Table 2:** Exclusion criteria

**Condition**	**Exclusion criteria**
Present disease or disease history	Autoimmune disease Inflammatory bowel diseases Past major abdominal or pelvic surgery
Therapy	Immune-modulating or immunosuppressive Antibiotics therapy up to three months before enrolment
Feeding-related conditions	Usage of tube feeding Known or suspected allergy to any treatment or placebo component
Cognitive impairment	Known and established cognitive decline Any comorbidity preventing reliable completion of trial assessments
Parkinson’s symptoms	Motor fluctuations

Patients will be required to report any proposed modification in their medical therapy. Patients who will need substantial changes in antiparkinsonian therapy will be dropped from the trial. The use of laxative or prokinetic drugs will be recorded and is not an exclusion criterion per se, but the introduction of these medications or posology variations should be avoided during the trial.

Since the trial is primarily aimed at confirming the immunomodulating properties of probiotic treatment, any intercurrent and symptomatic infections, inflammatory episodes, or other medical conditions will be recorded. Evaluation by clinical staff for potential patient dropout will be based on the severity and temporal proximity of the event to scheduled visits and blood withdrawal. All participants will be advised to maintain a constant dietary intake and a regular physical activity during the trial. A daily report including quality of life aspects will be requested and evaluated using appropriate clinical scales. Informed consent will be obtained by clinicians after the eligibility assessment and during the first visit. All subjects or their legal representatives will be given all relevant information about the study structure and objectives. Additional consent will be requested for the use of collected data and biological specimens in ancillary studies.

#### Interventions

This is a placebo-controlled trial. The intervention group will be treated with a daily dose of probiotics, while the control group will be given identical packages containing maltodextrin only, used as a bulking agent in the probiotics’ formulation for the intervention group. In this pilot study, our aim is to assess the potential of probiotics therapy to modify the peripheral immune system profile and function in a cohort of PD subjects. In this pilot trial, the potential immunomodulating effects of probiotics will be tested. Placebo treatment was chosen as a comparator for this new approach to immunomodulation in PD.

During the enrolment visit, the medical and neurological examination will assess the need for immediate variations in medical therapy for each participant. Within 2 weeks, any therapy modification will be completed, and a baseline visit will be scheduled.

During the baseline visit (T0), physical and neurological examinations will be repeated to confirm the subject’s conditions and persistence of inclusion criteria, a baseline blood withdrawal will be performed, and all clinical evaluation scales will be completed to set baseline scores. Each participant, after the subscription of an appropriate informed consent, will be randomised and given a treatment box, containing single-dose sachets with 2.7g of powder of the allocated formulation. Patients will be instructed to take daily doses at home every morning before breakfast for 12 weeks, mixing the content of one sachet in about 125 ml of fresh water or other cold, non-carbonated drink. Participants will be instructed to keep unused or empty packaging for recollection and compliance evaluation.

Product compositions per dose (1 sachet, 2.7g) for the treatment and placebo compounds are listed in the following Table 3.

**Table 3:** Treatment and placebo composition

**Treatment composition**	
**Component**	**Quantity**
Bifidobacterium animalis subsp. lactis BS01	≥ 1 x 10^9^ CFU
Bifidobacterium longum 03	≥ 1 x 10^9^ CFU
Bifidobacterium adolescentis BA02	≥ 1 x 10^9^ CFU
Fructo-oligosaccharides FOS	2500 mg
Maltodextrin	q.s.
**Placebo composition**	
**Component**	**Quantity**
Maltodextrin	q.s.

Follow-up visits will be scheduled at 6 weeks after T0 (T1) and at 12 weeks after T0 (T2). At T1, physical and neurological examination will be repeated, and all clinical scales administered again, except for Bristol Stool Form Chart and MOCA. At T2, all assessments performed at T0 will be carried out again, including blood withdrawal.

Participants will be advised to report any suspected adverse reaction and new or worsening symptoms. Every report will be recorded, and a thorough evaluation will be performed by neurologists in the clinical staff in collaboration with the subject’s primary care doctor. Feedback will be provided to the participant as well as indications on continuing or stopping treatment administration. In accordance with the Declaration of Helsinki by the World Medical Association, every participant can freely request to be removed from the study at any moment. All participants will be requested to record daily treatment usage and keep empty packaging or unused doses for recollection after the trial completion. One courtesy call will be scheduled during treatment to reduce compliance issues. The use of immunomodulating drugs or antibiotic therapy is an exclusion criterion and patients starting such therapy during the trial period will be dropped from the study. Dose adjustments in antiparkinsonian therapy are permitted but should be avoided and substantial modifications to said therapies will cause dropping out from the study.

Every participant will be evaluated at enrolment by a clinical neurologist, who will assess needed adjustments in antiparkinsonian therapy that will be completed within two weeks from enrolment and before treatment administration to minimise the need for medical therapy modifications during the trial. The use of laxative or prokinetic drugs will be recorded and is not an exclusion criterion per se, but the introduction of these medications or posology variations should be avoided during the trial.

### Outcomes

#### Primary feasibility outcomes

To assess the feasibility of the proposed clinical trial, we defined different feasibility objectives, as previously listed in Table 1.

More in detail, we will evaluate the potential recruitment rate for the clinical trial by recording the number of eligible outpatients who scheduled a neurological examination in the proposed clinical setting during the pilot trial. The acceptance rate will also be evaluated, by recording the fraction of identified eligible subjects who will be willing to participate in the proposed clinical trial. Both assessments together will allow for an initial estimate of the needed duration for the main clinical trial, once an appropriate sample size will be defined based on the pilot trial results. During the pilot trial, the dropout rate will also be recorded to adjust the predicted trial duration based on the fraction of dropped-out subjects during the trial period.

Reported reasons for subject drop-out will also be recorded and further analysed to confirm the safety of both the treatment and placebo administration to enrolled subjects. Collected data during the pilot trial will be analysed to assess whether a significant variation in any recorded parameter will occur between the two trial branches. This will allow to accordingly select one or more appropriate primary outcomes for the main trial, among collected immune profile data. Secondary outcomes for the main trial will be selected in the same way, among parameters who will show a remarkable variation.

More in detail, in our previous in vitro study (Magistrelli et al., 2019), we demonstrated that probiotics modulate ROS and cytokine production by driving an anti-inflammatory profile. Therefore, we will test whether these properties are confirmed in vivo. Particularly, in our study, we want to analyse whether probiotics may modulate cytokines production, such as IFN-γ, TNF-α, IL-4, IL-10, and ROS production.

In order to investigate possible changes in immune phenotype, we plan to deepen the profile of both innate and adaptive immunity by means of a cytofluorimetric evaluation, according to the strategy described by Kustrimovic et al. (2018) and with additional panels specifically dedicated to innate immunity. The following cell subsets of the adaptive immune system will be assessed: CD4+ and CD8+ T naïve/memory cells, CD4+ T helper subsets (Th1, Th2, and Th17), and CD4+ regulatory T cells (conventional, naïve, and activated Treg). Moreover, for innate immunity, monocytes (classical, non-classical, and intermediate), and NK cells (NK CD56 dim and CD56 bright) will be also assessed. We will compare pre-treatment and post-treatment analysis and compare our intervention group with the placebo group at T0 and T2.

#### Patient-centred outcomes

A significant variation in scores resulting from appropriate questionnaires and scales will be evaluated as an outcome measure for exploratory and patient-centred endpoints (variations in motor and non-motor symptoms from T0 to T1 and T2).

### Participant timeline

The timeline of the proposed study is summarised in the following Table 4. Each patient will be visited within 2 weeks before starting the proposed treatment to assess eligibility and collect informed consent. Treatment with probiotics or placebo will then be started (T0) and carried out for 12 weeks, when the administration will be stopped and final assessments will be performed (T2). An additional interim assessment is scheduled at 6 weeks after T0 (T1).

**Table 4:** Participant timeline

**Visit**	Enrolment	T0	T1	T2
Time Point	Within 2 weeks before T0	T0	6 weeks after T0	12 weeks after T0
Informed consent	X			
Physical/neurological examination	X	X	X	X
Vital signs	X	X	X	X
Unified Parkinson’s disease rating scale (UPDRS)		X	X	X
Hoehn and Yahr assessment (H&Y)		X	X	X
Zung self-rating anxiety scale		X	X	X
Beck depression inventory scale (BDI-II)		X	X	X
Composite autonomic symptoms scale-31 (COMPASS 31)		X	X	X
Montreal cognitive assessment (MOCA)		X		X
PAC-QOL		X	X	X
Non-motor symptoms scale (NMSS)		X	X	X
Wexner scale		X	X	X
Constipation assessment scale (CAS)		X	X	X
Bristol stool form chart		X		X
Blood withdrawal		X		X
Treatment/placebo delivery		X		

### Sample size

The design of this pilot study is explorative and equally randomised to treatment with probiotics or placebo. Due to the exploratory nature of the proposed study, a formal sample size calculation is not strictly required [18]. In our precedent in vitro study, statistically significant results were found in a small cohort of 40 PD patients for all tested probiotic strains, with a global reduction in proinflammatory cytokines production and an increase in anti-inflammatory cytokines. Doubling this sample size, with at least 80 specimens to be collected at both T0 and T2, will allow us to correctly evaluate the feasibility of the future main study.

We thus used data from the said study to estimate sample sizes for the in vitro effect of all tested probiotic strains and a sample size of 80 subjects allows for the determination of most tested cytokines variations with an expected power greater than 80%, setting our threshold for statistical significance at 0.05.

As previously stated, the drop-out rate of this pilot study will also be recorded and used to determine an appropriate sample size increase for the main study.

### Recruitment

Recruitment will occur in Azienda Ospedaliero-Universitaria Maggiore della Carità di Novara. Enrolment will be proposed to eligible subjects by clinical neurologists during routine visits and given their recorded consent to receive information about ongoing research and recruiting trials. Patients will be recruited continuously until reaching the desired sample size and will start the trial period asynchronously. The recruitment period will be limited in time to avoid seasonal diet modifications as a confounding factor for some of the trial outcomes.

### Assignment of interventions

#### Allocation

Participants will be assigned to the two intervention arms using the minimisation method, with a 1:1 allocation ratio. Codes for the two arms of the study will be different, but investigators, clinical, and laboratory staff will not know which code is assigned to the placebo or intervention group. This assignation method will minimise predictable differences between the two groups (e.g., sex, age). Packages for the treatment formulation and placebo will be identical and although treatment and placebo packages will be differently coded, participants, clinicians, and all laboratory operators will be blinded to the coding scheme.

#### Blinding

Trial participants, care providers, outcome assessors, and data analysts will be blinded to treatment allocation. Only the provider of the treatment and placebo packages will know the coding schemes for the corresponding packages and will not disclose this information until after trial completion.

No unblinding procedure is needed. Participants will be advised to report any suspected adverse reaction and new or worsening symptoms. Every report will be recorded, and a thorough evaluation will be performed by clinical staff. Feedback will be provided to the participant as well as indications on continuing or stopping treatment administration, without unblinding the subject’s allocation.

### Data collection and management

#### Plans for assessment and collection of outcomes

Collected data will include clinical scales and questionnaires, administered by trained clinical neurologists at baseline visit T0, visit T1, and visit T2, and data from analyses carried out by trained technicians and laboratory staff on faecal samples and whole blood samples collected at T0 and T2.

Blood specimen processing protocols are available from the corresponding author upon request. Follow-up visits will be scheduled at 6 weeks after T0 (T1) and at 12 weeks after T0 (T2). At T1, physical and neurological examination will be repeated, and all clinical scales administered again, except for Bristol Stool Form Chart and MOCA. At T2, all assessments performed at T0 will be carried out again, including blood withdrawal.

At T0 and T2, withdrawal of 40 ml venous blood will be performed after a fasting night, between 8:00 and 10:00 am, in EDTA-coated tubes (BD Vacutainer). Tubes will be subsequently coded and stored at room temperature until processing, which will occur within 24 h after collection. A complete blood count with differential analysis will be conducted on separate blood samples.

### Brief description of protocols for endpoint assessment

#### Plasma cytokine measurement

The possible influence of probiotic treatment on the inflammatory profile will be evaluated by measuring at T0 and T2 the plasma levels of pro (e.g., TNF-α, IFN-γ) and anti-inflammatory cytokines (IL-10, IL-4). To this end, at each visit time, plasma aliquots from every sample will be separated and stored for cytokines assays (ELISA Ready-SET-Go! Affymetrix eBioscience, USA). Two milliliters of fresh blood will be centrifuged at 1400g for 10 min at room temperature and two plasma aliquots of 350 µL each will be stored in 1.5mL vials to assay extracellular cytokines levels.

#### ROS production

The production of ROS will be assessed using the superoxide dismutase-sensitive cytochrome C reduction assay, as nmol of reduced cytochrome C/10^6^ cells/30 min, using an extinction coefficient of 21.1 mM. RPMI 1640 without phenol red and FBS will be used for incubating cells in order to avoid any spectrophotometric interference.

#### Flow cytometric evaluation of immune phenotype

For FACS assays of immune phenotype, 5 mL of the peripheral venous blood will be collected, and 100 or 150 µL aliquots of whole blood samples will be incubated with a cocktail of anti-human antibodies (ab) specific for each evaluated cell subset. For naïve-memory cell subset, samples will be stained with anti-human CD45, CD3, CD4, CD8, CD45RA, and CCR7 ab for the identification of naïve (CD45RA+CCR7+), central memory (Tcm, CD45RA-CCR7+), effector memory (Tem, CD45RA-CCR7-), and effector memory RA+ (Temra, CD45RA+CCR7-) on CD3+CD4+ and CD3+CD8+ lymphocytes. CD4+ T helper subsets will be assessed by staining the whole blood sample with a cocktail of anti-human CD45, CD3, CD4, CXCR3, CCR4, and CCR6 ab for the identification of Th1 cells (CD3+CD4+CXCR3+CCR4-CCR6-), Th2 cells (CD3+CD4+CXCR3-CCR4+CCR6-), Th17 cells (CD3+CD4+CXCR3-CCR4+CCR6+), and Th1-Th17 cells (CD3+CD4+CXCR3+CCR4-CCR6+).

Total CD4+ regulatory T cells (cTreg, CD3+CD4+CD25highCD127low) and naïve (nTreg, CD3+CD4+CD25highCD127lowCD45RA+), activated (aTreg, CD3+CD4+CD25highCD127lowCD45RA-) Treg will be evaluated by incubating whole blood with the following ab: CD45, CD3, CD4, CD25, CD127, and CD45RA. The aliquot of the blood for the evaluation of monocytes will be incubated with a cocktail of anti-human CD45, HLA-DR, CD14, and CD16 ab for the identification of classical (HLA-DR+CD14highCD16-), non-classical (HLA-DR+CD14lowCD16high), and intermediate (HLA-DR+CD14highCD16+) monocytes. Finally, another aliquot of whole blood samples will be stained with a cocktail of anti-human CD45, CD3, CD56, and CD57 for the identification of total NK cells (CD3-CD56+) and their two main subsets: CD56dim (CD3-CD56dimCD16bright) and CD56bright (CD3-CD56brightCD16dim), together with the evaluation of the maturation marker CD57. The acquisition will be performed on a BD FACS Celesta flow cytometer (Becton Dickinson Italy, Milan, Italy) with BD FACS Diva software (version 8.0.1.1) and data will be analysed with FlowJo software (version 10.7.1).

#### Transcriptional factors mRNA evaluation

According to our previous studies (Kustrimovic et al., 2018; De Francesco et al., 2021), we aim to investigate whether probiotic treatment is also able to modify the mRNA levels of the main transcriptional factors in CD4+ T lymphocytes. To this end, CD4-positive cells will be obtained by PBMC, which will be separated from whole blood using Ficoll-Paque Plus density gradient centrifugation. After resuspension, any residual contaminating erythrocytes will be lysed by the addition of 10 mL of lysis buffer ((g/L) NH4Cl 8.248, KHCO_3_ 1.0, EDTA 0.0368). Cells will be washed twice in PBS by the addition of 10 mL of PBS, then centrifuged at 300 g for 10 min at RT and resuspended in 10 mL of RPMI/10% FBS. A manual cell count will then be performed to set CD4 separation reagent quantities. Typical PBMC preparations will contain at least 80% lymphocytes. CD4^+^ T cells will then be isolated from PBMC by means of Dynabeads CD4-Positive Isolation kit. At least 50,000 separated CD4^+^ T cells will then be resuspended in PerfectPure RNA lysis buffer (5 Prime GmbH, Hamburg, Germany), and the total RNA will be extracted by PerfectPure RNA Cell Kit™. Reverse-transcription will be performed on the resulting mRNA using a random primer and a high-capacity cDNA RT kit. Real-Time PCR reactions will be performed with 1 μM cDNA. Amplification of cDNA will allow for the analysis of mRNA levels of the transcription factor genes TBX21, STAT1, STAT3, STAT4, STAT6, RORC, GATA3, FOXP3, and NR4A2.

#### Motor and non-motor symptoms, quality of life monitoring

The clinical evaluation will be completed by the administration of forms and questionnaires. In particular, every participant will fill out the forms and questionnaires listed in the following Table 5 at T0, T1, and T2. Data from these scales will be used to monitor motor and non-motor symptoms and perceived quality of life during the trial.

**Table 5:** Clinical scales for the assessment of motor and non-motor symptoms and quality of life monitoring

**Clinical scale**	**Reference (DOI)**
Unified Parkinson’s disease rating scale (UPDRS)	10.1002/mds.10473
Hoehn and Yahr assessment (H&Y)	10.1002/mds.20213
Zung self-rating anxiety scale	10.1016/S0033-3182(71)71479-0
Beck depression inventory scale (BDI-II)	10.1002/mds.20792
Composite autonomic symptoms scale-31 (COMPASS 31)	10.1016/j.mayocp.2012.10.013
Montreal cognitive assessment (MOCA)	10.1590/1980-57642018dn13-010008
Patient assessment of constipation (PAC-QoL)	10.1080/00365520510012208
Non-motor symptoms scale (NMSS)	10.1111/ane.13336
Wexner scale	10.1007/BF02056950
Constipation assessment scale (CAS)	10.12968/ijpn.2012.18.7.321

### Plans to promote participant retention and complete follow-up

Participants will be contacted by phone to timely schedule T1 and T2 visits. Any intercurrent health issue will be recorded and evaluated by clinical neurologists, who will assess the need to drop the subject from the study protocol. Any previously collected data will be kept after dropout and used as appropriate in statistical analyses.

### Data management and confidentiality

Data for all participants will be recorded by clinical staff at the enrolment location at scheduled visits. Every entry will be double-checked by at least two different investigators and data will be monitored upon collection to evaluate the consistency and promote early identification of abnormal outliers.

Personal information about trial participants will be strictly confidential and managed by clinical staff at the enrolment location. Investigators who will manage collected specimens and perform data analyses will be blinded to the subjects’ identity. Every collected sample will be assigned a unique code, which will be used by all non-clinical investigators for specimen identification. Clinical staff will ensure the confidentiality of participants’ data.

### Plans for collection, laboratory evaluation, and storage of biological specimens for genetic or molecular analysis in this trial/future use

All laboratory analyses will be executed after storage of specimens at −80°C, performing batches of sample analyses. Every sample in storage journals will be identified with an anonymised code.

### Statistical methods

#### Statistical methods for outcome evaluation

To assess the main trial feasibility, the drop-out rate will be reported as the absolute number and percentage of total participants for both the intervention and placebo arms and will be accounted for when assessing an appropriate sample size for the main study. Feasibility will also be evaluated by recording all drop-out reasons and accordingly estimating an increase of the foreseen sample size.

Collected results will be reported as mean ± standard deviation (SD) of the mean, or median ± 25th and 75th percentile, as appropriate, and *n*, with *n* indicating the number of observations. The normality of data distribution will be analysed by means of D’Agostino-Pearson omnibus normality test. Mann–Whitney *U* test, Wilcoxon test, and Kruskal-Wallis tests will be used to assess differences in the distribution of non-parametric data. Paired and unpaired *t* tests will be used, as appropriate, to compare means and standard deviations for parametric data in the two treatment groups. Wilcoxon signed ranks test will be used to compare measures taken at T0 with the corresponding measures at T1 and T2 in the assessment of primary and secondary outcomes. When appropriate, efficacy outcomes will be reported as estimates with 95% confidence intervals. Statistical significance for correlations will be set at *p*<0.05. Commercial software (GraphPad Prism version 9.3.0 for Windows, GraphPad Software, San Diego, CA, USA, www.graphpad.com) will be used for data analyses and graph plotting.

#### Interim analyses

No interim analyses will be performed on collected data. Enrolment in the study will proceed sequentially, and each participant will immediately start administration of the assigned intervention, so data from each participant will be collected independently and even a partial dataset will not be available for interim analyses until the trial will be in an advanced stage of completion. No subgroup or other additional analyses will be performed. Non-adherent patients and dropouts will be excluded from the study and related data will not be collected.

The full protocol and used and/or analysed during the current study will be provided by the corresponding author upon request.

### Oversight and monitoring

#### Composition of the coordinating centre and trial steering committee

Trial management on a day-to-day basis will be carried out by clinical staff at the coordinating centre and by staff at the Center for Research in Medical Pharmacology. Neurological and clinical assessment, enrolment, and data collection will be performed by neurologists, postgraduate students in neurology, and medical students at the trial centre. Postgraduate students in Clinical Pharmacology and Toxicology and staff at the Center for Research in Medical Pharmacology will receive collected data and manage data storage at the University of Insubria, where data analysis will be performed by academic staff.

The trial steering committee will include academic staff from the University of Insubria and the University of Piemonte Orientale, who will monitor recruitment and timely data collection, oversee results reporting and publication. The committee will meet on a regular basis to monitor the regular course of the trial and ensure successful completion.

#### Composition of the data monitoring committee, its role, and reporting structure

The institutional review board explicitly approved the described trial protocol, for which the appointment of a Data Monitoring Committee was not requested, as no interim analyses will be performed besides subjective questionnaires. Appropriate safety monitoring will be performed directly on a per-subject basis by clinical staff. Furthermore, this pilot study protocol specifically excludes subjects with unstable or critical clinical conditions, and treatment with placebo or probiotics, during a relatively short period, is not expected to interfere with currently prescribed medical therapies.

#### Adverse event reporting and harms

All potential adverse events will be recorded by clinical staff and participants will be encouraged to report any new or worsening symptom, which will timely and thoroughly be evaluated for possible correlations with the administered treatment.

#### Frequency and plans for auditing trial conduct

No auditing will be performed for this trial.

#### Plans for communicating important protocol amendments to relevant parties (e.g., trial participants, ethical committees)

No protocol modifications are planned within the trial. Possible amendments will be communicated to the local ethics committee before the enrolment of participants.

## Discussion

The involvement of the immune system in the development and progression of Parkinson’s disease makes it a promising target for a disease-modifying therapy. This clinical trial will test the ability of probiotics therapy in modulating the peripheral immune system, which in turn is expected to have beneficial effects on disease progression and patients’ quality of life. If this trial shows a potential beneficial role of probiotics in immunomodulating peripheral immunity in PD patients, a trial with a larger cohort could be designed and disease progression could be monitored over a longer period to assess the disease-modifying potential of this intervention.

The possibility to use probiotics as adjuvant therapy in Parkinson’s disease could also help in reducing necessary drugs for the treatment of disease-related symptoms such as laxatives, prokinetics, and others (with a final reduction of individual therapy undesired effects and costs). The results of this study will also point further research in a disease-modifying direction. If further research shows a potential disease-modifying effect of probiotics, treatment could be proposed to all PD patients without any specific risks and increasing the quality of life in these patients.

In our mono-centric setting and considering rigorous exclusion criteria, one possible problem will be the recruitment of a large enough number of subjects. Enrolment will therefore be proposed to all eligible subjects by clinical neurologists during routine visits and given their recorded consent to receive information about ongoing research and recruiting trials. This will help in reaching the maximum number of possible participants, while ensuring strict adherence to inclusion/exclusion criteria, since participation will be only proposed to eligible patients by their neurologist and after a thorough medical evaluation.

All participants will be contacted during the trial to assess adherence to the assigned treatment and prevent dropouts whenever possible. Considering the current pandemic by SARS-CoV-2 and related vaccination campaigns, we will also record the vaccination status of all participants, so as to identify any possible interactions with clinical and laboratory data gathered for this trial. Since every participant will start the trial in a different time frame, we plan to schedule each participant’s agenda to minimise interactions with possible vaccine administrations or medical therapy variations. Such information will be gathered and, if needed, used at the best of our possibilities to assess potential biases in randomisation or data analyses.

## Trial status

This protocol is based on the protocol published on ClinicalTrials.gov with ID: NCT05173701 and unique protocol ID: PROB-PD. The last update on said protocol was submitted on 18 February 2022. Recruitment for the trial began on 22 November 2021, and 40 subjects have already been recruited at the time of this writing.

## Data Availability

The PI of this study and all involved investigators will have access to the final dataset. No contractual agreements will limit such access for any investigator.

## Abbreviations

PD: Parkinson’s disease
α-syn: α-Synuclein
ROS: Reactive oxygen species
IFN: Interferon
TNF: Tumour necrosis factor
IL: Interleukin
PBMC: Peripheral blood mononuclear cell
TGF: Transforming growth factor
NK: Natural killer
ab: Antibodies
